# Retraction: Neuroprotective Role of L-N^G^-Nitroarginine Methyl Ester (L-NAME) against Chronic Hypobaric Hypoxia with Crowding Stress (CHC) Induced Depression-Like Behaviour

**DOI:** 10.1371/journal.pone.0341205

**Published:** 2026-01-21

**Authors:** The PLOS One Editors

After this article [1] was published, the following concerns were raised:

- The following panels appear to partially overlap:
  - Fig 7C Control+Veh and Fig 7C Control+L-NAME
  - Fig 7C Control+L-NAME and Fig 7D Control+L-NAME
  - Fig 10A CHC+Veh and Fig 10B CHC+Veh
- There are concerns about undeclared competing interests during peer review.

In response to the concern for Fig 7C Control+Veh and Fig 7C Control+L-NAME, the first author stated that the Fig 7C Control+Veh panel in [1] is incorrect. An updated Fig 7C Control+Veh panel was provided by the first author along with the original data underlying Fig 7. PLOS considers this concern to be resolved.

In response to the concerns for partial overlap between Fig 7C Control+L-NAME and Fig 7D Control+L-NAME, and between Fig 10A CHC + Veh and Fig 10B CHC + Veh, the first author stated that these panels represent two different regions of the hippocampus (CA1 and CA3). They stated that overlap is visible between the panels in question as they were captured from the same sections, and due to the close proximity of these regions. They also stated that with image stack overflow, there is expected to be a certain degree of overlap between images. Regarding the effect of these partial overlaps between Figs 7C and 7D and between Figs 10A and 10B on the corresponding quantitative data in Figs 7E-G, and Figs 10D-F respectively, the first author stated that the regions selected for quantification were delineated using anatomical landmarks and standardized field-of-view coordinates using blind scoring protocols, to avoid any overlap in image analysis. They also stated that each group consisted of 20 animals (n = 20), and that for each animal, 10–15 coronal sections spanning the septal hippocampus were analyzed. They stated that the quantified values per animal were averaged across the multiple sections, and the group mean (± SEM) was then calculated across the 20 animals. Image data for all 20 subjects were not provided during post-publication discussions and the first author stated that most of the primary data underlying this article [1] are no longer available.

During editorial follow-up, a member of the *PLOS One* Editorial Board reviewed the above concerns and the responses received, and stated that although it is common practice in image stack processing to have a certain degree of overlap between images, the above overlap within Fig 7 and within Fig 10 in [1] comprises extensive overlapping regions of interest that includes both the CA1 and CA3 regions of the hippocampus, and that image stack processing is employed when image stitching is performed. Neither image stack processing nor image stitching are described in [1]. Given that the discrepancy between the response to the partial overlap concerns between Fig 7C Control+L-NAME and Fig 7D Control+L-NAME, and between Fig 10A CHC + Veh and Fig 10B CHC + Veh. and the methods described in [1], this issue remains unresolved.

Due to the concerns with peer review, another member of the *PLOS One* Editorial Board reviewed this article [1] and noted the following:

- There are concerns regarding the statistical analysis results, for example
  - In the Crowding stress data in Fig 2A, although the change is greater on day 21 compared to day 3, this does not appear to be reflected in the corresponding p-values.
  - For tests involving three or more groups, post-hoc tests were performed after conducting an analysis of variance but it is not possible to determine which values correspond to comparisons between which groups.
- There are concerns regarding the immunohistochemical staining results
  - Some images do not reflect the quantitative results. Specifically, in Figs 4A and 4B, the numbers of positive cells and arrows appear similar. However, in Fig 4C, the number of positive cells differs significantly enough between CA1 and CA3 regions to detect a statistical difference.
  - In Figs 4D and E, counterstaining with DAPI is not shown and therefore it is unclear how the cell count was quantified. In addition, since the arrows point to signals, it is difficult to determine whether they indicate individual cells.
  - In Fig 6, it is unlikely that such a high number of nuclear aggregation cells could be observed, including in the control group. In addition, the areas labelled as vacuolation are observed in large numbers in surrounding tissues such as CA1 which may indicate freezing damage to the cells during sample preparation. It is concerning that cells thought to be observed under these specific conditions (where cell death is induced by chemical substances or stress exposure) are readily observed in the control group as such cells are rarely seen in untreated groups.
  - In Fig 7, some brain tissue is oriented to the right and some to the left which raises concerns that differences may exist between the right and left hemispheres. It has not been demonstrated in [1] that there is no time for the distribution of molecules used for comparison, that there is no difference between the right and left brain hemispheres in the control group or that mixing the right and left brain hemispheres does not influence the results.
  - In Fig 9, no caspase-3 staining signal can be confirmed as the background is too strong. In addition, although images showing microglial phagocytosis are presented, it is difficult to distinguish them which raises concerns with the associated quantitative data.

In light of the above concerns which remain unresolved in the absence of much of the underlying data and that question the reliability of the reported results, the *PLOS One* Editors retract this article.

SND did not respond to the final editorial decision. IB, AS, AKSG, DP, and SBS did not respond directly or could not be reached.
